# Bacterial Survey in the Guts of Domestic Silkworms, *Bombyx mori* L.

**DOI:** 10.3390/insects13010100

**Published:** 2022-01-17

**Authors:** Ivan Y. Dee Tan, Ma. Anita M. Bautista

**Affiliations:** 1 Research and Development Division, Philippine Textile Research Institute, Department of Science and Technology, Bicutan, Taguig City 1631, Philippines; iydeetan@upd.edu.ph; 2 National Institute of Molecular Biology and Biotechnology, University of the Philippines—Diliman, Quezon City 1101, Philippines

**Keywords:** 16S rRNA gene, amplicon sequencing, *Bombyx mori*, gut microbiota

## Abstract

**Simple Summary:**

To enhance the sustainability of commercial production of high-quality silk, factors that affect the economic characteristics of the silkworm and the silk it produces have been widely studied. Among these are the gut microbiota, which have been linked to absorption and utilization of nutrients, and immunity to diseases in silkworms. Because the Philippines has yet to improve the silkworm strains it currently uses for silk production, sufficient biological data, including that of microbiota, are warranted. Profiling the bacterial communities in local silkworm strains through the use of high-throughput 16S rRNA gene amplicon sequencing would be a source of useful information. Results of the 16S rRNA gene amplicon sequencing in this study showed that four of the silkworm strains reared in the Philippines are abundant in five bacterial genera, which have also been found in other silkworm strains. Results also showed that bacterial diversity and evenness increase as larvae mature, which can be correlated to larval development and to the shift in the amount and age of mulberry leaves the larvae consume.

**Abstract:**

Silkworm, *Bombyx mori* L., research involves studies on improving strains for enhanced sustainability of high-quality silk production. Several of these have investigated the factors affecting growth and development of silkworm larvae and cocoon characteristics that subsequently affect the yield and quality of silk. The gut microbiota has been reported to impact growth and development of silkworms and has been linked, in particular, with absorption and utilization of nutrients and immunity to diseases. The silkworm strains maintained in the Philippines lack sufficient biological data for use in strain improvement. This prompted efforts to augment the data by profiling bacterial communities through high-throughput 16S rRNA gene amplicon sequencing and analysis in four of the local silkworm strains that are bred and maintained in the country. Results of the study showed that the four silkworm strains are abundant in bacteria that belong to the genera *Pseudomonas*, *Sphingomonas*, *Delftia*, *Methylobacterium* and *Acinetobacter*. Results also showed that bacterial diversity and evenness increase as larvae mature, which can be correlated to larval development and shifts in the amount and age of mulberry leaves the larvae consume.

## 1. Introduction

*Bombyx mori* L., commonly referred to as the domestic silkworm [1] or mulberry silkworm [2], feeds exclusively on the leaves of the mulberry plant, *Morus alba* [3]. Belonging to the family Bombycidae [4], the insect is known for its ability to produce silk fibers through the formation of its cocoon [5]. *Bombyx mori* has been reared for as long as 5000 years [4] and has become central to the silk production industry [6]. *Bombyx mori* is also the model organism representative of the order Lepidoptera [7]. It is next to *Drosophila melanogaster* in terms of the number of genetic studies conducted on insects [4].

Countries in Asia remain the top producers of natural silk [8], contributing to 98% of the world’s supply [2]. High demand for silk in the textile and fashion industries globally prompts sericulturists to improve the production of silk and enhance its quality [9]. Recent efforts to increase the capacity for silk production include breeding silkworms with improved economically important traits. In the Philippines, key qualitative traits used to assess strains for the production of silk include size, weight, and shape of cocoons, hatchability of fertilized eggs, larval development time, larval and pupal weight, moth emergence, reproductive capacity of moths, and mortality rate [10]. Of these, strains exhibiting faster larval development, accompanied by increased cocoon weight and yield are the most preferred. The Philippine silk industry, however, has been facing issues with the quality of silkworms, and these contribute to low cocoon production [11]. The local silk industry, thus, needs silkworms with improved quality. Genetic characterization of the 17 strains that are reared, bred, and maintained in germplasm centers in the country has already been initiated by Alcudia-Catalma et al. 2020 [9], but additional biological information is deemed necessary.

The growth and development of silkworm larvae and the economic characteristics of cocoons are influenced largely by nutritional and climatic factors, rearing techniques, silkworm races, and other factors [12]. Like most insects, silkworms also harbor a consortium of microorganisms that play crucial roles in their survival [13]. The presence of symbiotic bacteria in their gut, for example, allows for the absorption and utilization of nutrients and provides immunity to diseases [14]. These affect silkworm growth, development, and survival, which in turn affect the yield and quality of cocoons. Microbial community investigation can be performed through approaches that utilize high-throughput next generation sequencing techniques. 16S rRNA gene amplicon sequencing is one approach that enables the identification of species of microorganisms present in environmental samples and provides information on the function of these microorganisms through the analysis of genomic data. This approach offers a way to bypass the challenge posed by bacterial species that cannot be cultured because it employs analyses to investigate bacterial diversity, abundance, processes, and possible interactions of an entire bacterial community based on genetic content of samples collected [15]. The process by which 16S rRNA gene amplicon sequencing allows “direct access” to the genetic information of various microorganisms from the environment [16] relies on the 16S rRNA gene conserved in prokaryotic organisms [17]. Bacterial species may be identified based on the sequences of this gene [17]. More recently, next-generation sequencing has dramatically sped up this process through different platforms that allow for sequencing of target regions in multiple samples, assembly of multiple reads into contiguous sequences, matching sequences to either a single target organism or to related species, and ultimately assigning the organisms to the selected taxonomic level [16].

The main goal of the present study was to profile and compare the bacterial communities found in the guts of Philippine silkworm strains and their larval instars using 16S rRNA gene amplicon sequencing. Results from this study provide sources of information that are associated with the target local strains that have been reared, bred, and maintained in the germplasm centers in the Philippines for decades [9], but biological data remain scarce to enhance commercial sustainability. The data generated here can be used in strain improvement through conventional hybridization and selective breeding, which is widely practiced in other silk-producing countries for enhanced sustainability of silk production.

## 2. Materials and Methods

### 2.1. Sample Collection, Preservation, and Selection

Four *B. mori* strains were considered for inclusion in this study. These silkworm strains (K203, MO202, MO204, and DMMMSU 119) were reared at the Philippine Textile Research Institute Technology Center in Misamis Oriental (PTRI-TCMO) and were chosen for analysis based on the available data describing each strain’s larval development, as well as their cocoon and silk characteristics (Table S1). Based on the facility’s characterization of strains and on the report of Basaen et al. (1982) [10], high hatch percentage, short larval duration, high cocoon weight, and high silk yield are considered as the most economically important and preferred silkworm traits.

All test strains were reared within the same facility under similar conditions and fed with mulberry leaves collected from the same field. However, early instars (first to third) were fed with young leaves, whereas later instars (fourth and fifth) were fed with fully developed leaves according to the practices of the facility. The second, third, fourth, and fifth instars of each of the silkworm strains were preserved in 95% ethanol and stored at 4 °C prior to gut dissection and genomic DNA extraction. Ethanol preservation was adopted based on Hammer et al. (2015) [18].

### 2.2. Silkworm Gut Dissection and DNA Extraction

One fifth instar larva, one fourth instar larva, three third instar larvae, and five second instar larvae from each strain were first washed with distilled water to remove excess ethanol used for preservation. These were then dissected on the dorsal side to extract the guts, which were washed with distilled water to remove excess plant material within. For the second and third instars, the guts of individuals belonging to the same strain were pooled to obtain 10–30 mg of starting material for DNA extraction. Two biological replicates were made in total using guts of different larvae from the same batch of eggs. Dissection and pooling of silkworm guts was done inside a laminar flow hood. Sterile scalpel blades and forceps used for dissection were also changed in between the dissection of each strain and instar.

DNA was extracted from the guts using the Quick-DNA Tissue/Insect Miniprep Kit (Zymo Research, Irvine, CA, USA) following the manufacturer’s instructions. The extracted DNA was quantified using Qubit dsDNA BR assay (Invitrogen, Waltham, MA, USA). The quality and purity of the extracts was assessed using 1% agarose gel electrophoresis and Nanodrop spectrophotometer (Scientific Industries, Bohemia, NY, USA), respectively. DNA extracts were stored at −40 °C prior to library preparation.

### 2.3. Library Preparation and Sequencing on MiSeq FGx

Libraries were prepared and pooled from a total of 32 samples (4 silkworm strains × 4 instar stages × 2 biological replicates) based on the *16S* Metagenomic Sequencing Library Preparation guide by Illumina with a few modifications. DNA extracts were diluted to 10 ng/µL using nuclease-free water. Primers 341F and 805R containing 5′ overhang sequences (Table S2) used for *16S* V3-V4 PCR were from Herlemann et al. (2011) [19]. The amplicon PCR mix contained the following: 2.5 µL DNA template (15 ng/µL), 5 µL primer 341F (6 µM), 5 µL primer 805R (6 µM), and 12.5 µL MyFi PCR mix (Bioline Reagents Ltd., United Kingdom) for a total reaction volume of 25 µL. PCR was then performed under the following conditions: initial denaturation at 95 °C for 3 min; 35 cycles of denaturation at 95 °C for 30 s, annealing at 55 °C for 30 s; extension at 72 °C for 30 s; final extension at 72 °C for 5 min; and held at 4 °C.

Pairs of different indices (index 1 and index 2) from the Nextera XT Index kit (Illumina, San Diego, CA, USA) were selected such that each sample would have a unique pair of indices, as presented in Table S3. The index PCR mix contained the following: 5 µL amplicon PCR product, 5 µL Nextera XT index primer 1, 5 µL Nextera XT index primer 2, 25 µL MyFi PCR Mix, and 10 µL nuclease-free water, for a total reaction volume of 50 µL. PCR was done under the following conditions: initial denaturation at 95 °C for 3 min; 8 cycles of denaturation at 95 °C for 30 s; annealing at 55 °C for 30 s; extension at 72 °C for 30 s; final extension at 72 °C for 5 min; and held at 4 °C.

Individual library concentrations in nM were calculated based on Qubit concentration (ng/µL) and amplicon size based on TapeStation (Agilent, Santa Clara, CA, USA).

Calculated library concentrations were then normalized, pooled, and denatured. Pre-chilled HT1 hybridization buffer was then added to dilute the denatured library to 20 pM. The PhiX control (Illumina, San Diego, CA, USA) library was prepared following the above procedure. A final concentration of 8 pM was prepared with 15% PhiX spike-in. The combined library was loaded into the MiSeq v3 (600 cycles, 2 × 300 bp reads) reagent cartridge (Illumina, San Diego, CA, USA), and the sequencing run using the MiSeq FGx (Illumina, San Diego, CA, USA) was initiated.

### 2.4. 16S rRNA Amplicon Sequence Analysis

The summary of the sequencing run is detailed in Table S4. The percent Q30 score of the run was 93.0%, which is above the minimum of 70% recommended by Illumina. Raw DNA sequence data generated were deposited in the NCBI Sequence Read Archive (SRA) under the accession numbers listed in Table S5.

Sequence data were then converted to FASTQ format and used for downstream analyses using QIIME 2 version 2021.11 (Available online: https://qiime2.org/ (accessed on 2 January 2022)). The QIIME 2 workflow is summarized in Figure S1. Lengths of 300 bp were obtained for the paired-end reads, and raw sequence counts per sample ranged from 32,620 to 80,412 sequences. Quality control was performed using DADA2 (Available online: https://qiime2.org/ (accessed on 2 January 2022)) included in the QIIME 2 pipeline. The final sequence counts per sample, which ranged from 13,700 to 41,059 after quality filtering, denoising, merging forward and reverse reads, and removing chimeric sequences using DADA2 prior to QIIME 2 analysis, are detailed in Table S6. This allows for replicate sequences to be collapsed into representative sequences (rep-seqs) used for classification. The range of the final sequence counts of this study fell within the range reported by Suenami et al. (2019) [20] (i.e., 12,627 to 56,357 for QIIME 2 analysis of the gut microbiome of hornets).

The representative sequences generated were then used for naïve Bayes taxonomic classification using the Silva 138 classifier, which calculates the probability that a sequence belongs to a certain species instead of sequence alignment [21]. Classification using QIIME 2 in this study, therefore, did not require a percent identity threshold value. Sequences of mitochondria and chloroplasts were then filtered out prior to diversity analyses. A sampling depth of 2420 was used for diversity analyses. This value was used such that all samples and the most sequences possible would be included in the study. This sampling depth would enable the analysis of the majority of the silkworm gut microbiome as the generated alpha-rarefaction plot showed that most sequence counts begin to taper off at this point and sample count declines past this point (Figure S2). Kruskal–Wallis and PERMANOVA tests (999 permutations) were conducted to determine the significance of alpha-diversity and beta-diversity analyses, respectively. A heatmap showing the abundance of bacterial genera across all samples was also generated (Figure S3).

The feature table that was subsequently generated after rep-seqs generation by QIIME 2 was then used to determine the most frequently observed representative sequences across samples. These rep-seqs were used for BLAST queries of bacteria that were associated with each of the genera. NCBI BLAST queries were performed using the nt database on 10 January 2022. The top BLAST hits for the sequences corresponding to the five most abundant bacterial taxa according to QIIME 2 classification were determined.

## 3. Results

### 3.1. Analysis of Bacterial Abundance

**Figure 1:**
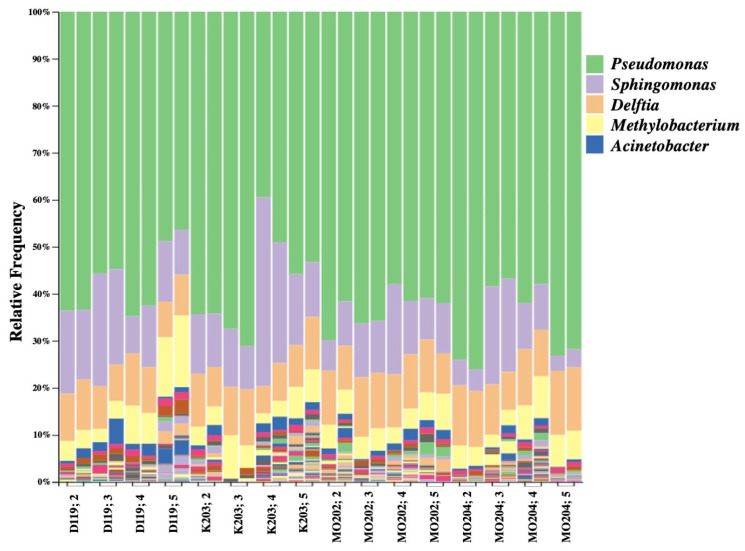
QIIME 2 bar plot of the top five most abundant bacterial genera per instar of each *Bombyx mori* L. strain based on two biological replicates using Silva classifier (top five genera: *Pseudomonas*, *Sphingomonas*, *Delftia*, *Methylobacterium, Acinetobacter*).

Bacterial genera were identified based on abundance by QIIME 2 using the Silva classifier. Among the top five most abundant genera, *Pseudomonas* appeared to be the most dominant genus across all silkworm strains and instars (Figure 1). It displayed lower abundance percentages in later instars of DMMMSU 119 (fifth instar) and K201 (fourth and fifth instars), but was still ranked the most abundant. *Sphingomonas* and *Delftia* were typically ranked the second to fourth most abundant in most of the strains and instars. These are followed by *Methylobacterium* which was generally ranked third to fifth most abundant across all instars and strains. *Acinetobacter* was ranked the fourth to fifth most abundant bacterial genus overall.

NCBI BLAST queries (Table S7) revealed that the most frequent representative sequences under each of the genera were species that have been isolated from various sources, including soil, plant, and insect gut [22]. BLAST queries also returned hits for contaminating uncultured and cultured bacteria that might have come from the reagents or the laboratory used to prepare the sequencing templates and libraries.

### 3.2. Alpha-Diversity Analysis

**Figure 2:**
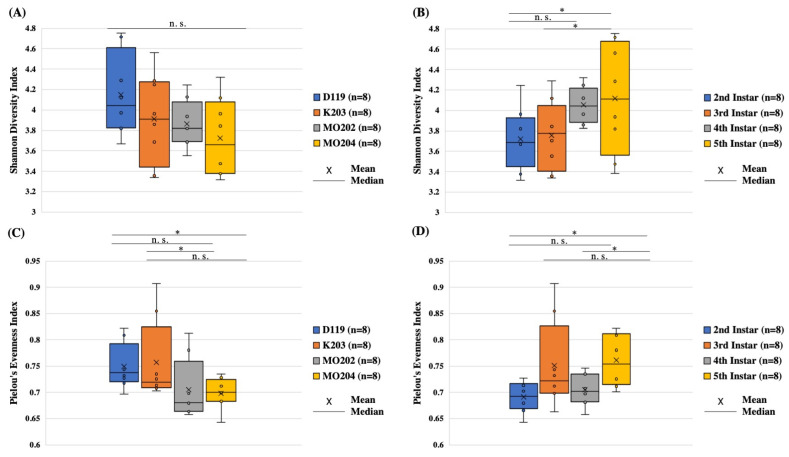
Alpha-diversity plots for *Bombyx mori* L. strains and instars reared at the Philippine Textile Research Institute Technological Center in Misamis Oriental (PTRI-TCMO). (**A**) Shannon diversity index boxplot for *Bombyx mori* L. strains; (**B**) Shannon diversity index boxplot for *Bombyx mori* L. instars; (**C**) Pielou’s evenness index boxplot for *Bombyx mori* L. strains; (**D**) Pielou’s evenness index boxplot for *Bombyx mori* L. instars. Significance (*p* < 0.05) is indicated with a (*). Comparisons that are not significant (p > 0.05) are indicated with “n.s.”

Bar plots detailing Shannon diversity index values per silkworm strain and instar are presented in Figure 2A,B, respectively. Individual Shannon diversity values for each strain and instar are listed in Table S8. Silkworm strains do not seem to differ significantly in terms of diversity based on *p*-values from Kruskal–Wallis tests for all groups (Table S9). Strain MO202 possesses the closest levels of bacterial diversity among its instars (Figure 2A). This strain, however, also appears to be the second least diverse on average compared to the other strains. DMMMSU 119 appears the most diverse on average compared to other strains, followed by K203 as the second most diverse strain. The individual instars of DMMMSU 119, K203, and MO204 appear to have different diversity values, suggesting that some instars of each strain are rich in the number of bacterial taxa within their guts and some instars have low bacterial diversity. MO204 has the lowest mean Shannon diversity index value, indicating that this strain is the least diverse on average.

Silkworm instars also do not appear significantly different in terms of diversity based on *p*-values from Kruskal–Wallis tests for all groups (Table S9). Among the silkworm instars across the four silkworm strains, the fifth instars seem to be the richest in terms of bacterial taxa, followed by the third instars (Figure 2B). Fourth instars, and subsequently the second instars, appear less diverse on average. Only fourth instars appear to differ significantly from second instars (*p* = 0.0117) and third instars (*p* = 0.0460), however. These significant differences in terms of bacterial diversity in the guts of some silkworm instars may correlate to the increase in mulberry leaf consumption and the potential changes in the gut bacterial community as a result. Chen et al. (2018) [3] also reported a shift in abundance of particular bacteria in the guts of silkworms from early to late instar stages.

Bar plots detailing Pielou’s evenness index values per silkworm strain and instar are presented in Figure 2C,D, respectively. Individual Pielou’s evenness values for each strain and instar are listed in Table S10. Silkworm strains appear to differ significantly in terms of evenness based on *p*-values from Kruskal–Wallis tests for all groups (Table S11). Strain K203 appears to be the most even in terms of the distribution of bacterial taxa on average (Figure 2C). DMMMSU 119 and MO204 appear as the second and third most even strains in terms of bacterial taxa distribution. DMMMSU 119 was also significantly different from MO204 in terms of evenness (*p* = 0.0209). The ranking of mean evenness values for DMMMSU 119 and K203 differs from those of their mean diversity values wherein DMMMSU 119 was more diverse than K203 (Figure 2A). MO202 and MO204 appear less even on average (Figure 2C), similar to their lower mean Shannon diversity values (Figure 2A).

Silkworm instars did not seem to differ significantly in terms of evenness based on *p*-values from Kruskal–Wallis tests for all groups (Table S11). Similar to the trend in diversity, fifth instars appear to be the most even in terms of bacterial taxa distribution (Figure 2D). Fifth instars only appeared significantly different from second instars in terms of evenness (*p* = 0.0117), however. Unlike the trend in diversity, third instars appeared more diverse than fourth instars (Figure 2D). Second instars were still the least even on average, however (Figure 2D), which is similar to their ranking as the least diverse instar on average (Figure 2B). Second and fourth instars were also more similar in terms of evenness within their respective groups compared to third and fifth instars. The trend of evenness values displayed by the instars matches that of their Shannon diversity index values (Figure 2B), suggesting that evenness possibly contributed to their diversity. This may also suggest that when fewer dominant bacterial taxa are present, there would be more taxa overall due to the absence of certain taxa that monopolize the environment and resources, thus, preventing others from thriving.

### 3.3. Beta-Diversity Analysis

**Figure 3:**
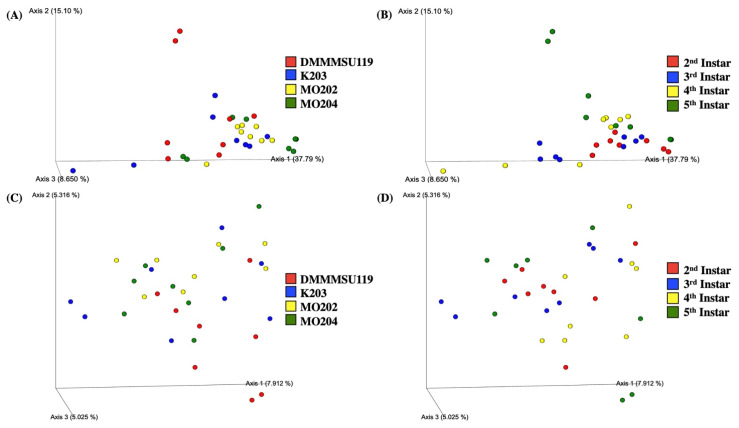
Beta-diversity plots for *Bombyx mori* L. strains and instars reared at the Philippine Textile Research Institute Technological Center in Misamis Oriental (PTRI-TCMO). (**A**) Bray–Curtis dissimilarity sorted by *Bombyx mori* L. strains (*p* = 0.03); (**B**) Bray–Curtis dissimilarity sorted by *Bombyx mori* L. instars (*p* = 0.048); (**C**) Jaccard similarity index sorted by *Bombyx mori* L. strains (*p* = 0.079); (**D**) Jaccard similarity index sorted by *Bombyx mori* L. instars (*p* = 0.024).

Bray–Curtis dissimilarity 3D plots sorted by silkworm strain and instars are shown in Figure 3A,B. Silkworm strains appeared to differ significantly in terms of Bray–Curtis dissimilarity based on *p*-values from PERMANOVA tests (999 permutations) for all groups (Table S12). Figure 3A shows similar bacterial abundance within instars of strain MO202. Instars of strains DMMMSU 119, K203, and MO204 appear more dissimilar to other instars of their respective strain. Strain DMMMSU 119 also appears the most dissimilar compared to the other three strains, but only significantly different from strains MO202 (*p* = 0.004) and MO204 (*p* = 0.021). Silkworm instars also appeared significantly different in terms of Bray–Curtis dissimilarity based on *p*-values from PERMANOVA tests (999 permutations) for all groups (Table S12). Based on the Bray–Curtis dissimilarity values of the instars, diversity in second instars appears to be the most similar group and only varies significantly from the fourth instars (*p* = 0.029) (Figure 3B). Although there appears to be dissimilarity among the different instars, these are not significant when compared in a pairwise manner.

The Jaccard similarity index 3D plots sorted by silkworm strain and instar stages are shown in Figure 3C,D. Silkworm strains did not appear significantly different from each other in terms of Jaccard similarity based on *p*-values from PERMANOVA tests (999 permutations) for all groups (Table S13). Figure 3C shows no clustering within strains; however, the values are relatively close to one another. This further suggests that silkworm strains are similar in terms of the bacterial taxa that are present and absent (not considering the abundance of the taxa that are present). Despite the similarities observed, strain DMMMSU 119 appeared significantly different from strains MO202 (*p* = 0.018) and MO204 (*p* = 0.002) once again. Silkworm instars, in contrast, appeared significantly different from one another in terms of Jaccard similarity based on *p*-values from PERMANOVA tests (999 permutations) for all groups (Table S13). Once again, no clustering was observed within any of the instars. The values all appeared relatively similar to each other. Only the third and fifth instars appeared significantly different from one another (*p* = 0.042). Combined with the generally low Bray–Curtis dissimilarity values for most strains and instars, this suggests that the common bacterial taxa found among silkworm strains and instars possess close abundance values.

## 4. Discussion

### 4.1. Bacterial Abundance

The dominant bacterial communities associated with Philippine *B. mori* belong to the genera *Pseudomonas*, *Sphingomonas*, *Delftia*, *Methylobacterium,* and *Acinetobacter*. These genera have been associated with mulberries [23,24,25] and were also found prevalent in the guts of other strains of silkworms [3,17]. This indicates shared similarities between silkworms reared in the Philippines and silkworms reared elsewhere, in terms of the bacterial genera present in their guts. However, these bacterial communities still differ in terms of overall genera that are present as well as their relative abundance.

For the species identity of bacteria under each genus, NCBI BLAST queries revealed that the most frequent representative sequences under *Pseudomonas* were mostly identical to *Pseudomonas fluorescens* strains, which were isolated either from sediments or from gut of adult fruit-tree pinhole borer, *Xyleborinus saxesenii,* an ambrosia beetle. Like several bacteria of the genus *Pseudomonas*, the *P. fluorescens* group is found in diverse environments and well-known for its plant-beneficial properties, including pathogen suppression [26]. Thus, the *Pseudomonas* species found in silkworms in this study likely originated from mulberries and from the field where these host plants were cultivated. However, there were recent observations that some strains of this group can also colonize insects and cause severe infections leading to death [27]. *Pseudomonas* strains from other studies also showed amylolytic, cellulolytic, xylanolytic, lipolytic, and esterase activity in silkworms and beetles [28,29,30]. Hence, the sequence similarity of *Pseudomonas* rep-seqs in this study was notable in consideration of the ability to hydrolyze polymers of mulberries. BLAST queries also returned hits for uncultured bacterium isolated from a human body part, implicating the presence of contaminants. This could have been prevented to a certain extent if a negative control was included in the sequencing and analysis. BLAST also returned hits of most frequent rep-seqs for strains of *Sphingomonas* that were isolated from nectarine tree and paddy soil. Species of this genus have no reported biological function in the other strains of silkworm where they were detected, but some species have been observed to possess the ability to degrade organic matter [31] and also the ability to produce beta-carotene and gellan gum [32,33]. *Sphingomonas* has also been associated with diapause-destined female of the cabbage beetle, *Colaphellus bowringi* [34] and was found to be one of the genera of cellulolytic bacteria in the gut of the Chinese white pine beetle, *Dendroctonus armandi*, larvae [35]. Species of *Sphingomonas* have also been reported to be part of the core members of mulberry endophytes regardless of the season or cultivar [36]. Contaminating *Sphingomonas* species from sterile water was also detected, however, highlighting further the need for controls in this type of study.

*Delftia* species isolated from the gut of another moth and from soil were also among the BLAST hits for rep-seqs in this study. Besides finding *Delftia* strain in silkworms in other studies, a recent report indicated its presence in diapause-destined *C. bowringi* [34]. A species of *Delftia* (*D. acidovorans*) isolated from the American cockroach, *Periplaneta americana* L., which was found to produce bacteriocins against bacterial and fungal pathogens of humans [37] was also recently reported.

Functions linked to the presence of bacteria under the genus *Methylobacterium* include nitrogen-fixing in silkworm guts [3] and cellulolytic activity in the gut of the Chinese white pine beetle, *D. armandi*, larvae [35]. BLAST hits for *Methylobacterium* in the present study points to strains that might have been involved in nitrogen-fixation and -removal because the strains with hits were isolated from the rhizosphere and sewage sludge undergoing nitrogen removal. The strain similar to that isolated from a fish gut has no associated function, however.

*Acinetobacter* strains from other studies have shown amylolytic, cellulolytic, xylanolytic, lipolytic, and esterase activity in silkworms and beetles [28,29,30], but the strains most frequent in the silkworms used in this study were highly similar to those isolated from goose stools and soil.

Of interest in the data presented in this study is the relevance of these dominant bacterial communities in Philippine silkworm biology, which remains to be explored further.

### 4.2. Alpha-Diversity and Beta-Diversity

The increase in average bacterial diversity as the larvae mature suggests a shift in gut bacterial diversity. This occurs either in response to host development or an increase in leaf consumption and the age of mulberry leaves that were used. Strains that possessed high Shannon diversity values also possessed high Pielou’s evenness values. This may suggest a potential link between bacterial diversity and evenness among the silkworm strains. This also indicates that bacterial diversity and evenness may be related given that the presence of fewer dominant bacterial taxa may allow more taxa to grow, as it is less likely that a certain taxon would monopolize resources or inhibit the growth of others. It should be noted, however, that neither silkworm strains nor instars appeared significantly different in terms of bacterial diversity overall, and only silkworm strains were significantly different in terms of bacterial evenness. This again relates to the similarity of the strains in terms of phenotypic traits and the most abundant bacterial taxa in their guts. These are also expected as the strains are genetically similar [9].

Quantitative measures of similarity among strains (Bray–Curtis dissimilarity) seem to set strain DMMMSU 119 apart from the other three silkworm strains. The other strains cluster together supporting the observed similar phenotypic traits. Whereas silkworm instars appeared significantly different from one another overall, only second and fourth instars appeared significantly different from each other. Both strains and instars appear similar in terms of qualitative measures of similarity that do not take bacterial abundance into account (Jaccard similarity). DMMMSU 119 was also significantly different from strains MO202 and MO204, and third and fifth instars also appeared significantly different from each other. This indicates that strains and instars are similar in terms of the bacterial taxa present but are less similar in terms of the actual abundance of these bacterial taxa.

## 5. Summary and Conclusions

In this study, the bacterial communities of the four silkworm strains reared in the Philippines were investigated using high-throughput 16S rRNA gene amplicon sequencing and analysis. The results showed the abundance of five bacterial genera that were also found in other silkworm strains. Although the likely association of some of the bacterial species to biological activity in the gut as well as their involvement in the growth, development, and survival of silkworms have been reported, the exact roles of the bacteria within the silkworm strains in the present study and how these roles correlate to the production economically important silk characteristics. remain to be elucidated. Evidently, the results expand the biological data available for the silkworm strains present in the country and will be a useful source of information for future strain improvement to enhance the sustainability of quality silk production.

## Data Availability

The raw DNA sequence data generated and used in this study are available on NCBI’s Sequence Read Archive (SRA) with accession numbers SRR17009362–SRR17009393.

## Supplementary Materials

The following supporting information can be downloaded at: https://www.mdpi.com/article/10.3390/insects13010100/s1: Table S1: Traits of *Bombyx mori* L. strains for selection, Table S2: Primer and Illumina adapter overhang sequences for *16S* V3-V4 PCR, Table S3: Nextera XT index pair assignments for each *16S* library prepared, Table S4: Sequencing run summary, Table S5: SRA accession numbers for *Bombyx mori* L. samples, Table S6: Sequence count summary for each *Bombyx mori* L. sample, Table S7: Representative sequences corresponding to top bacterial genera, their frequencies, top BLAST hits, and isolation sources, Table S8: Shannon’s diversity boxplot values for each *Bombyx mori* L. strain and instar, Table S9: Comparison of Shannon’s diversity index *p*-values, Table S10: Pielou’s evenness boxplot values for each *Bombyx mori* L. strain and instar, Table S11: Comparison of Pielou’s evenness index *p*-values, Table S12: Comparison of Bray–Curtis dissimilarity *p*-values, Table S13: Comparison of Jaccard similarity *p*-values, Figure S1: Diagram of QIIME 2 workflow, Figure S2: Alpha-rarefaction curves of each *Bombyx mori* L. strain, Figure S3: QIIME 2 heatmap of bacterial genera across the *Bombyx mori* L. samples.

## References

[1] Mita K., Kasahara M., Sasaki S., Nagayasu Y., Yamada T., Kanamori H., Namiki N., Kitagawa M., Yamashita H., Yasukochi Y. (2004). The genome sequence of silkworm, *Bombyx mori*. DNA Res..

[2] Mohanta M.K., Saha A.K., Saleh D.K.M.A., Islam M.S., Mannan K.S.B., Fakruddin M. (2010). Characterization of *Klebsiella granulomatis* pathogenic to silkworm, *Bombyx mori* L.. Biotech.

[3] Chen B., Du K., Sun C., Vimalanathan A., Liang X., Li Y., Wang B., Lu X., Li L., Shao Y. (2018). Gut bacterial and fungal communities of the domesticated silkworm (*Bombyx mori*) and wild mulberry-feeding relatives. ISME J..

[4] Nagaraju J., Goldsmith M.R. (2002). Silkworm genomics—Progress and prospects. Curr. Sci..

[5] Xia Q.Y., Zhou Z.Y., Lu C., Cheng D., Dai F.-Y., Liu B., Zhao P., Zha X., Cheng T., Biology Analysis Group (2004). A draft sequence for the genome of the domesticated silkworm (*Bombyx mori*). Science.

[6] Pereira N.C., Munhoz R.E.F., Bignotto T.S., Bespalhuk R., Garay L.B., Saez C.R.N., Fernandez M.A. (2013). Biological and molecular characterization of silkworm strains from the Brazilian germplasm bank of *Bombyx mori*. Genet. Mol. Res..

[7] Zanatta D.B., Bravo J.P., Barbosa J.F., Munhoz R.E.F., Fernandez M.A. (2009). Evaluation of economically important traits from sixteen parental strains of the silkworm *Bombyx mori* L. (Lepidoptera: Bombycidae). Neotrop. Entomol..

[8] Matienzo L.H., Gatica R.A. Sericulture: Silk Production in the Philippines. https://aboutphilippines.org/files/Silk-Production-in-the-Philippines.pdf.

[9] Alcudia-Catalma M.N., Conde M.Y.E.D., Dee Tan I.Y., Bautista M.A.M. (2020). First report on the characterization of genetic diversity of Philippine-reared *Bombyx mori* strains based on *COI* and ITS2. Philipp. J. Sci..

[10] Basaen A.M., Placido A., Bacuso P., Ladines A., Cabrito F. (1982). Silkworm breeding for the development of Philippine purelines. NSTA Technol. J..

[11] Villanueva R.C. (1999). Problems and Issues Affecting the Pace of Sericulture Development in the Philippines. https://agris.fao.org/agris-search/search.do?recordID=PH2007M00034.

[12] Sarkar K. (2018). Management of nutritional and climatic factors for silkworm rearing in West Bengal: A review. Int. J. Agric. Environ. Biotechnol..

[13] Barretto D.A., Gadwala M., Vootla S.K. (2021). Chapter 1—The silkworm gut microbiota: A potential source for biotechnological applications. Methods Microbiol..

[14] Sun Z., Kumar D., Cao G., Zhu L., Liu B., Zhu M., Liang Z., Kuang S., Chen F., Feng Y. (2017). Effects of transient high temperature treatment on the intestinal flora of the silkworm *Bombyx mori*. Sci. Rep..

[15] National Research Council (US) (2007). Committee on Metagenomics: Challenges and Functional Applications. The New Science of Metagenomics: Revealing Secrets of Our Microbial Planet.

[16] Thomas T., Gilbert J., Meyer F. (2021). Metagenomics—A guide from sampling to data analysis. Microb. Inform. Exp..

[17] Sun Z., Lu Y., Zhang H., Kumar D., Liu B., Gong Y., Zhu M., Zhu L., Liang Z., Kuang S. (2016). Effects of BmCPV infection on silkworm *Bombyx mori* intestinal bacteria. PLoS ONE.

[18] Hammer T., Dickerson J., Fierer N. (2015). Evidence-based recommendations on storing and handling specimens for analyses of insect microbiota. PeerJ.

[19] Herlemann D.P.R., Labrenz M., Jürgens K., Bertilsson S., Waniek J.J., Andersson A.F. (2011). Transitions in bacterial communities along the 2000 km salinity gradient of the Baltic Sea. ISME J..

[20] Suenami S., Nobu M.K., Miyazaki R. (2019). Community analysis of gut microbiota in hornets, the largest eusocial wasps, *Vespa mandarinia* and *V. simillima*. Sci. Rep..

[21] Wang Q., Garrity G., Tiedje J., Cole J. (2007). Naïve Bayesian classifier for rapid assignment of rRNA sequences into the new bacterial taxonomy. Appl. Environ. Microbiol..

[22] Schoch C.L., Ciufo S., Domrachev M., Hotton C.L., Kannan S., Khovanskaya R., Leipe D., McVeigh R., O’Neill K., Robbertse B. (2020). NCBI Taxonomy: A comprehensive update on curation, resources and tools. Database.

[23] Ou T., Xu W.-F., Wang F., Strobel G., Zhou Z.-Y., Xiang Z.-H., Liu J., Xie J. (2019). A microbiome study reveals seasonal variation in endophytic bacteria among different mulberry cultivars. Comput. Struct. Biotechnol. J..

[24] Lukša J., Servienė E. (2020). White mulberry (*Morus alba* L.) fruit-associated bacterial and fungal microbiota. J. Environ. Eng. Landsc. Manag..

[25] Dong H.L., Zhang S.X., Chen Z.H., Tao H., Li X., Qiu J.F., Xu S.Q. (2018). Differences in gut microbiota between silkworms (*Bombyx mori*) reared on fresh mulberry (*Morus alba* var. multicaulis) leaves or an artificial diet. RSC Adv..

[26] Flury P., Aellen N., Ruffner B., Péchy-Tarr M., Fataar S., Metla Z., Maurhofer M. (2016). Insect pathogenicity in plant-beneficial pseudomonads: Phylogenetic distribution and comparative genomics. ISME J..

[27] Flury P., Vesga P., Péchy-Tarr M., Aellen N., Dennert F., Hofer N., Kupferschmied K.P., Kupferschmied P., Metla Z., Ma Z. (2017). Antimicrobial and insecticidal: Cyclic lipopeptides and hydrogen cyanide produced by plant-beneficial *Pseudomonas* strains CHA0, CMR12a, and PCL1391 contribute to insect killing. Front. Microbiol..

[28] Prem Anand A.A., Vennison S.J., Sankar S.G., Gilwax Prabhu D.I., Vasan P.T., Raghuraman T., Vendan S.E. (2010). Isolation and characterization of bacteria from the gut of *Bombyx mori* that degrade cellulose, xylan, pectin and starch and their impact on digestion. J. Insect. Sci..

[29] Briones-Roblero C.I., Rodríguez-Díaz R., Santiago-Cruz J.A., Zúñiga G., Rivera-Orduña F.N. (2017). Degradation capacities of bacteria and yeasts isolated from the gut of *Dendroctonus rhizophagus* (Curculionidae: Scolytinae). Folia Microbiol. (Praha).

[30] Delalibera I., Handelsman J., Raffa K.F. (2005). Contrasts in cellulolytic activities of gut microorganisms between the wood borer, *Saperda vestita* (Coleoptera: Cerambycidae), and the bark beetles, *Ips pini* and *Dendroctonus frontalis* (Coleoptera: Curculionidae). Environ. Entomol..

[31] Gong B., Wu P., Huang Z., Li Y., Dang Z., Ruan B., Kang C., Zhu N. (2016). Enhanced degradation of phenol by *Sphingomonas* sp. GY2B with resistance towards suboptimal environment through adsorption on kaolinite. Chemosphere.

[32] Silva C., Cabral J.M.S., van Keulen F. (2004). Isolation of a beta-carotene over-producing soil bacterium, *Sphingomonas* sp.. Biotechnol. Lett..

[33] Wang X., Xu P., Yuan Y., Liu C., Zhang D., Yang Z., Yang C., Ma C. (2006). Modeling for gellan gum production by *Sphingomonas paucimobilis* ATCC 31461 in a simplified medium. Appl. Environ. Microbiol..

[34] Liu W., Li Y., Guo S., Yin H., Lei C., Wang X. (2016). Association between gut microbiota and diapause preparation in the cabbage beetle: A new perspective for studying insect diapause. Sci. Rep..

[35] Hu X., Yu J., Wang C., Chen H. (2014). Cellulolytic bacteria associated with the gut of *Dendroctonus armandi* larvae (Coleoptera: Curculionidae: Scolytinae). Forests.

[36] Xu W., Wang F., Zhang M., Ou T., Wang R., Strobel G., Xiang Z., Zhou Z., Xie J. (2019). Diversity of cultivable endophytic bacteria in mulberry and their potential for antimicrobial and plant growth-promoting activities. Microbiol. Res..

[37] Amer A., Hamdy B., Mahmoud D., Elanany M., Rady M., Alahmadi T., Alharbi S., AlAshaal S. (2021). Antagonistic activity of bacteria isolated from the *Periplaneta americana* L. gut against some multidrug-resistant human pathogens. Antibiotics.

