# Supplementary figures and images for: Bacterial Survey in the Guts of Domestic Silkworms, Bombyx mori L
Source: Insects. 2022 Jan 17;13(1):100. doi: 10.3390/insects13010100 (PMC8779307; doi:10.3390/insects13010100)

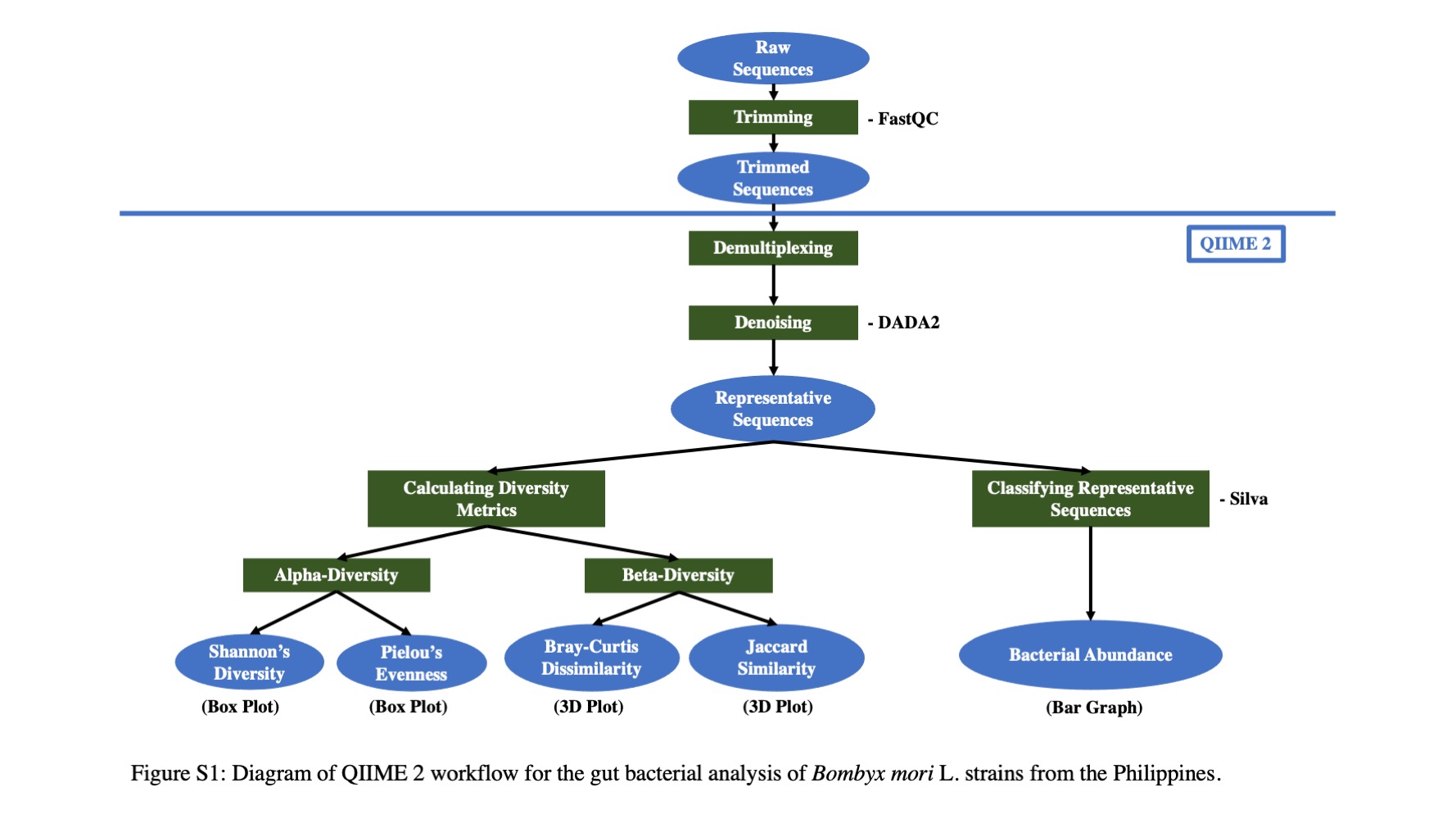

Supplement: Supplementary file 1 [file insects-13-00100-s001.zip › Figure S1 QIIME 2 workflow.jpg]

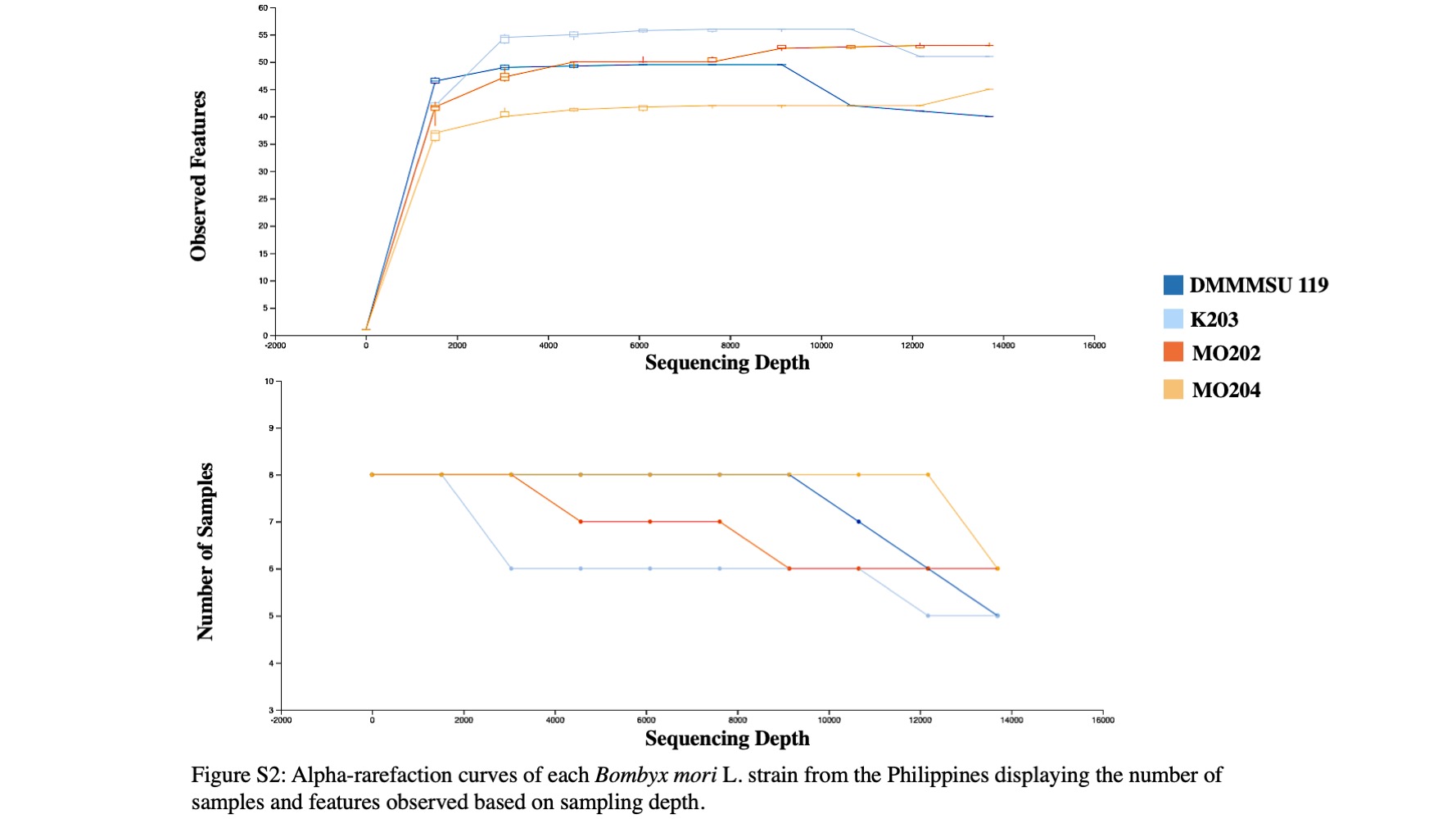

Supplement: Supplementary file 1 [file insects-13-00100-s001.zip › Figure S2 Alpha-rarefaction curves.jpg]
