# Supplementary material for: Bacterial Survey in the Guts of Domestic Silkworms, Bombyx mori L
Source: Insects. 2022 Jan 17;13(1):100. doi: 10.3390/insects13010100 (PMC8779307; doi:10.3390/insects13010100)
Supplement: Supplementary file 1 [file Figure S3 Bacterial genera heatmap.pdf › Figure S3 Bacterial genera heatmap.pdf]

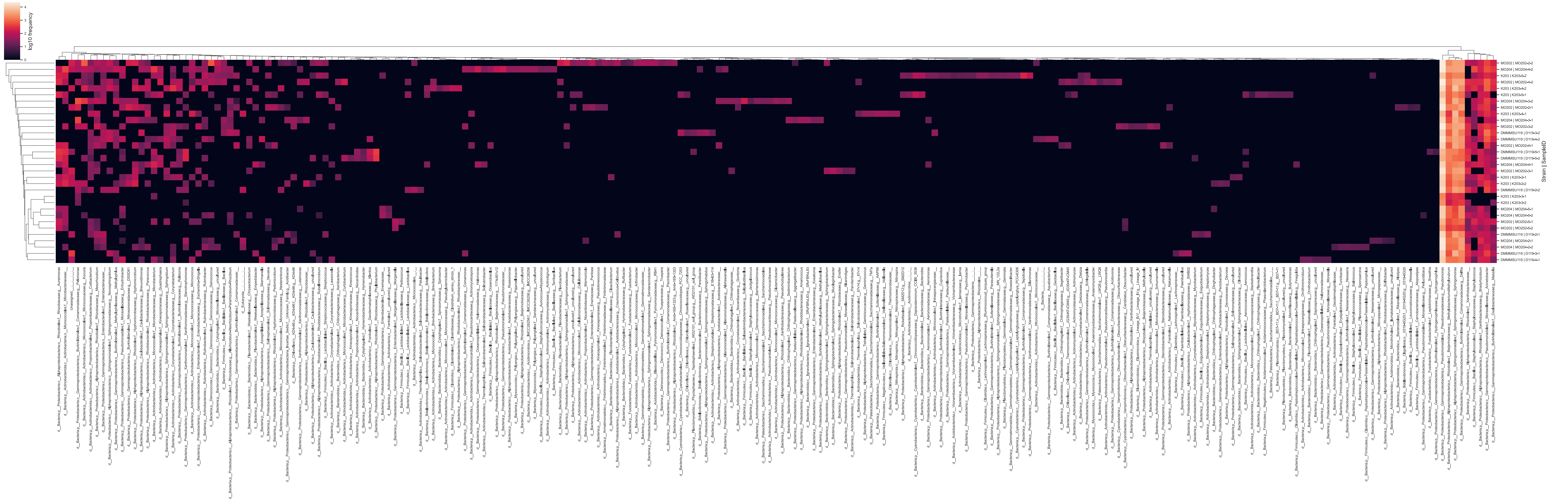

Figure S3. QIIME 2 heatmap of bacterial genera across *Bombyx mori* L. samples from the Philippines.
